# Developmental Dyslexia: Disorder or Specialization in Exploration?

**DOI:** 10.3389/fpsyg.2022.889245

**Published:** 2022-06-24

**Authors:** Helen Taylor, Martin David Vestergaard

**Affiliations:** ^1^Hunter Centre for Entrepreneurship, Strathclyde Business School, University of Strathclyde, Glasgow, United Kingdom; ^2^Department of Archaeology, Faculty of Human, Social and Political Science, School of the Humanities and Social Sciences, McDonald Institute for Archaeological Research, University of Cambridge, Cambridge, United Kingdom; ^3^Wolfson College, University of Cambridge, Cambridge, United Kingdom

**Keywords:** developmental dyslexia (DD), cognitive search, division and specialization, cultural evolution, adaptation, exploration – exploitation, complex adaptive system, individual learning

## Abstract

We raise the new possibility that people diagnosed with developmental dyslexia (DD) are specialized in explorative cognitive search, and rather than having a neurocognitive disorder, play an essential role in human adaptation. Most DD research has studied educational difficulties, with theories framing differences in neurocognitive processes as deficits. However, people with DD are also often proposed to have certain strengths – particularly in realms like discovery, invention, and creativity – that deficit-centered theories cannot explain. We investigate whether these strengths reflect an underlying explorative specialization. We re-examine experimental studies in psychology and neuroscience using the framework of *cognitive search*, whereby many psychological processes involve a trade-off between exploration and exploitation. We report evidence of an explorative bias in DD-associated cognitive strategies. High DD prevalence and an attendant explorative bias across multiple areas of cognition suggest the existence of explorative specialization. An evolutionary perspective explains the combination of findings and challenges the view that individuals with DD have a disorder. In cooperating groups, individual specialization is favored when features that confer fitness benefits are functionally incompatible. Evidence for search specialization suggests that, as with some other social organisms, humans mediate the exploration–exploitation trade-off by specializing in complementary strategies. The existence of a system of collective cognitive search that emerges through collaboration would help to explain our species’ exceptional adaptiveness. It also aligns with evidence for substantial variability during our evolutionary history and the notion that humans are adapted not to a particular habitat but to variability itself. Specialization creates interdependence and necessitates balancing complementary strategies. Reframing DD therefore underscores the urgency of changing certain cultural practices to ensure we do not inhibit adaptation. Key improvements would remove cultural barriers to exploration and nurture explorative learning in education, academia, and the workplace, as well as emphasize collaboration over competition. Specialization in complementary search abilities represents a meta-adaptation; through collaboration, this likely enables human groups (as a species and as cultural systems) to successfully adapt. Cultural change to support this system of collaborative search may therefore be essential in confronting the challenges humanity now faces.

## Introduction

At present, Developmental dyslexia (DD) is widely viewed as a neurobiological disorder (Valdois, 2010). The cognitive differences associated with DD were initially recognized because of lexical difficulties (Morgan, 1896). Later research has focused primarily on understanding the cognitive and neurophysiological processes that may explain these observed educational difficulties, particularly with reading and writing. Consequently, a literature examining the non-lexical effects experienced by individuals with DD is lacking (Tunmer and Greaney, 2010).

Developmental dyslexia is defined as “a disorder in children who, despite conventional classroom experience, fail to attain the language skills of reading, writing and spelling commensurate with their intellectual abilities” (World Federation of Neurology, 1968). Difficulty achieving a high level of reading and writing ability results from slow and inaccurate word recognition and spelling, problems that remain despite adequate instruction and intact sensory abilities (Peterson and Pennington, 2012).

This long-standing deficit-centric view provides an incomplete picture. Nearly forty years ago, Norman Geschwind noted an increasing number of studies suggesting that those with DD have superior talents in certain non-verbal skills that relate to art, architecture, engineering, and athletics. He was the first to highlight a likely evolutionary basis for the differences observed and, further, he suggested that when a relatively broad swath of a population exhibits a seemingly adverse condition, it is worth asking whether there might be some countervailing advantage at play (Geschwind, 1982). Decades on, researchers continue to ask similar questions, including Eide and Eide (2019), who noted: “[T]he question we need to be asking is not what’s wrong with the dyslexic brain, but what is dyslexic cognition for, what are these brains really built to do?.” Nevertheless, within academia there has been relatively little research or progress in understanding DD-associated abilities.

DD is understood to have a clear genetic basis (Valdois, 2010; Paracchini et al., 2016; see Erbeli et al., 2021b for a recent review). It is a complex trait involving multiple genes with twin studies indicating a heritability of at least 60% (Paracchini et al., 2016). Genetic influence on DD remains stable from adolescence to early adulthood, with the same genetic influences manifested across development from childhood to early adulthood (Wadsworth et al., 2007), suggesting that DD may not inherently be a disorder of development so much as a difficulty that is encountered during development.

The genetic basis is not the only good reason for taking an evolutionary perspective in attempting to understand DD-associated cognitive differences. DD also affects a large proportion of the population, between 5 and 20% (Badian, 1984; Wagner et al., 2020) and is universal and cross-cultural. Its cross-cultural nature suggests that the differences in cognition that underlie reading difficulties must have evolved by the emergence of behavioral modernity (ca. 150,000–50,000 BP).

The need to read and write is unlikely to have exerted any evolutionary selection pressure. Indeed, in the context of human existence, the technology of writing is a very recent invention (ca. 5350 BP) (Englund, 2004), with mass use among the general population only occurring over the last 100 years or so. It is the only example of a cultural invention for which we assume that difficulty in use relates to some kind of deficit: if someone does not show an aptitude for, say, accountancy or computer programming, we do not assume they have a neurobiological disorder.

Despite the recognition that there may be an evolutionary basis for DD (Geschwind, 1982; Stein, 2001), there has been no real attempt to explain DD-associated cognition from an evolutionary perspective until recently. A new evolutionary theory (Taylor et al., 2022) proposes that successful adaptation in humans arises from collaboration between individual members who are specialized in different but complementary neurocognitive search strategies (Taylor et al., 2022). This theory was developed to help explain DD-associated cognitive differences, by providing the theoretical understanding of why search specialization is likely to have evolved and its significance for understanding human adaptation and cultural evolution.

Here we reexamine DD-associated cognitive differences from the perspective of cognitive search – a theoretical approach applied to DD for the first time. Central to this is the understanding that many aspects of cognition can be viewed from the perspective of search, characterized by a trade-off between exploration and exploitation (Hills et al., 2015). We challenge the traditional view that the cognitive attributes of individuals with DD result from incorrect development. Instead, we propose that the features of this form of cognition were strongly selected for. It follows that what have been traditionally regarded as deficits are trade-offs, and that these are balanced by specialization and enhanced abilities in complementary areas of cognition.

This article does not intend to debate the existing evidence concerning DD nor understand why such individuals experience difficulties with writing technology. Rather, our aim is to reinterpret the existing evidence from the perspective of cognitive search and to understand the overall pattern of information processing in individuals with DD. Furthermore, the cognitive search paradigm provides a useful framework for generating hypotheses as to why these underlying cognitive differences may have evolved. When this new cognitive search perspective is combined with other lines of evidence, it is possible to see that the cognitive attributes identified in people with DD have a strong evolutionary imperative.

## Previous Approaches to Developmental Dyslexia

Throughout the history of dyslexia research, approaches have focused on identifying and remediating learning difficulties associated with dyslexia. Here, we outline key theories regarding the nature of DD-associated differences, including cognitive and biological theories.

In 1896, Pringle Morgan, a British physician, described a case of “congenital word-blindness” in an intelligent boy who had developmental difficulties with reading and spelling (Morgan, 1896). Whilst “word-blindness” had been described previously, Morgan was the first to postulate an underlying congenital problem (Kirby et al., 2020). Morgan (1896) assumed that “word-blindness” in his patients was caused by a visual processing deficit specific to words and spelling representations. This understanding remained the principle hypothesis on which dyslexia research was based over the next decades (e.g., Hinshelwood, 1917; Orton, 1925). However, DD readers were found to retain adequate perception of visual details, suggesting that there must be other underlying factors (Frith, 1978).

By the 1980s, the phonological deficit hypothesis emerged as the dominant theory of DD. The theory proposed that an underlying deficit in the representation, storage or retrieval of speech sounds impeded grapheme-phoneme mapping, resulted in difficulties in spelling and reading in alphabetic languages (e.g., Bradley and Bryant, 1978; Vellutino, 1979; Snowling, 1981; Stanovich, 1988; Brady and Shankweiler, 1991; Shaywitz and Shaywitz, 2005). Typical tasks where DD readers may struggle could include detecting whether words rhyme, deleting the initial or final phoneme, as examples. On a neurological level, differences in activation patterns in the perisylvian regions of the left hemisphere have been observed while participants were engaged in tasks involving phonological processing, inclusive of rhyme judgment, verbal working memory and pseudo-word reading (Démonet et al., 2004). While evidence supports the idea that phonological deficits contribute to the reading and spelling challenges of individuals with dyslexia, it cannot explain other differences associated with DD, such as differences in motor coordination.

A logical way to challenge the former approaches directly is to hypothesize that they are secondary to something else. Nicolson and Fawcett (1990) posited that, rather than being a specific difficulty with reading and writing, DD appears to be a more generalized learning difficulty: a difficulty with automatization, a skill that reduces the burden placed on working memory. This view holds that difficulties in acquiring and automatizing skills underlie the challenges observed over a range of areas, from fine motor skills to cognitive skills required to succeed in reading, writing, and mathematics. Given the perceived range of deficits associated with DD, including those involving motor skills and automatization, Nicolson and Fawcett identified the cortico-cerebellar circuit as a neurobiological basis of automaticity (Nicolson and Fawcett, 1990, 2007; Nicolson et al., 2001). Testing this across several studies, they found relative deficits in the awareness of time (Nicolson et al., 1995) and in postural stability and muscle tone: characteristics associated with cerebellar dysfunction (Fawcett and Nicolson, 1999). The delayed neural commitment theory further develops Nicolson and Fawcett’s automatization and cerebellar deficit hypothesis by considering the developmental process of constructing certain neural networks. The theory proposes that individuals with DD take longer to build and rebuild the neural networks that lie at the foundation of the kinds of skills discussed above, particularly those that are language-based (Nicolson and Fawcett, 2019).

The magnocellular deficit hypothesis provides another explanation of the deficits experienced in DD. Stein (2001, 2019) proposed that DD is “a hereditary temporal processing defect, associated with impaired magnocellular neuronal development” (Stein, 2018, 9). He outlined how temporal processing appears to be supported by magnocellular neurons, specialized in timing, that act in networks throughout the brain: in the cerebral cortex, hippocampus, cerebellum, and brainstem (Stein, 2018). These are involved in many functions including visual, auditory, touch, and proprioceptive systems. When magnocellular neurons are impaired, as is proposed to be the case in DD, several functions could be affected.

Until now, literature on the negative aspects of DD has dominated the field. Stein highlighted that the relatively high incidence of impaired development of the magnocellular system in individuals with DD “would not be so common unless there were compensating advantages for dyslexia” (Stein, 2001, 13). Like Stein, Nicolson and Fawcett have noted (Nicolson, 2014) that cognitive and neurophysiological theories regarding automatization may also help to explain some of the enhanced abilities observed in individuals with DD. They have suggested, for example, that the delay in automatization presents a trade-off in that conscious access to information is retained, making it easier to modify and integrate information and, in turn, to facilitate innovation (Nicolson, 2014).

While there are other approaches to DD that are not covered within the scope of this review, those discussed highlight some of the key approaches to visual and phonological deficit theories. Since they fail to identify a core deficit, key biological debates such as the automaticity/cerebellar and magnocellular approaches are also outlined. In general, however, the emphasis has been deficit-centric with few studies taking a more holistic approach that would incorporate both positive and negative differences experienced by individuals with DD.

Reflecting this, tools for identifying DD also tend to be deficit focused and may vary according to the approach of different practitioners. In general, the field has moved toward a multifactorial model of DD, whereby it is understood that most cases of DD cannot be explained by a single cognitive (or linguistic) “deficit” (Thompson et al., 2015). Relying on any one factor has been found to result in misclassification; for example, O’Brien and Yeatman (2021) found that relying on phonological processing measures alone lead to misclassification of DD in 30% of cases.

Multifactorial models recognize that a number of factors contribute to spelling, reading and writing difficulties experienced with DD. Some of the reported cognitive difficulties include rapid naming, phonological and morphological awareness, visual-orthographic knowledge, and verbal working memory (e.g., Shaywitz et al., 1999; Shaywitz and Shaywitz, 2005).

While most assessors consider a range of factors, assessments still vary between assessors and differences also exist between languages (Erbeli et al., 2021b). It is also important to note converging evidence from both twin studies and molecular genetic studies which indicate that reading ability lies on a continuum, with the cut-off point being arbitrary (Erbeli et al., 2021b). Taken together these factors may contribute to variation in reported forms of dyslexia and prevalence rates.

## Proposed Areas of Enhanced Ability Associated With Developmental Dyslexia

Although the above theories contribute to our understanding of DD-associated difficulties, the deficit-centered view does not tell the whole story. Observations that individuals with DD appear to exhibit countervailing advantages unexplained by current theories opens a new possibility: are there actual, one-sided deficits, or might the areas of difficulty be the downsides of trade-offs that exist, with the upsides being specialization and enhanced ability in other areas of cognition?

In order to determine whether individuals with DD are specialized to have a particular form of cognition, it is useful to consider whether the observed areas of enhanced ability share any fundamental pattern. In the rest of this section, we give a brief overview of such areas that are thought to be typical of people with DD. This is not a comprehensive or critical overview, as the lack of research on strengths precludes it. Rather, our aim is to point to areas of enhanced ability that have been proposed to exist by researchers and practitioners over the past four decades (e.g., Geschwind, 1982; West, 1997; Eide and Eide, 2011; Nicolson, 2014; Schneps, 2014).

Areas of enhanced ability that are consistently reported as being typical of people with DD include seeing the big picture, both literally and figuratively (e.g., von Károlyi, 2001; Schneps et al., 2012; Schneps, 2014), which involves a greater ability to reason in multiple dimensions (e.g., West, 1997; Eide and Eide, 2011). Eide and Eide (2011) have highlighted additional strengths related to seeing the bigger picture, such as the ability to detect and reason about complex systems, and to see connections between different perspectives and fields of knowledge, including the identification of patterns and analogies. They also observed that individuals with DD appear to have a heightened ability to simulate and make predictions about the future or about the unwitnessed past (Eide and Eide, 2011).

Individuals with DD have been proposed to exhibit greater ability in various areas of creativity. This has inspired several studies that have reported evidence for enhanced creative ability in a number of realms ranging from freeform drawing and other artistic objects (Cohn and Neumann, 1977) to literary creativity (Rack, 1981). Studies of creative ability also show evidence of a heightened ability to connect and carry out unusual combinations of ideas (Cancer et al., 2016), as well as heightened ability in tasks requiring novelty, insight, and more innovative styles of thinking (Everatt et al., 1999).

Practitioners have long observed that there appear to be high proportions of people with DD in professions and courses of study that rely on these abilities such as art and design, engineering, and entrepreneurship (see e.g., Geschwind, 1982; Martino and Winner, 1995; West, 1997; Newman and Sternberg, 2012).

In the realm of entrepreneurship, there has also been a growing interest in the apparently large numbers of entrepreneurs with DD (Alexander-Passe et al., 2021). A study of entrepreneurs in the United States found that 35% were dyslexic, with 22% being highly or extremely dyslexic (Logan, 2009).

In students enrolled in higher education, the incidence of DD is particularly high in creative subjects like arts and engineering. Wolff and Lundberg (2002) studied students enrolled at the University of Gothenburg, Sweden. They found that the prevalence of DD was significantly higher in students studying fine arts and photography compared with students studying economics and commercial law. At Central St Martins, University of the Arts London, United Kingdom, 75% of foundation year students had some form of DD (Steffert, 1996), and at the Royal College of Art, United Kingdom, 29% of students self-identified as having DD (RCA, 2015). In a study covering several United Kingdom universities across four degree disciplines (engineering, law, medicine, and dentistry), Lemon and Shah (2014) reported that self-identified DD in engineering was 28% compared with 5% in law. These self-reported figures are particularly high given that most people with DD do not get diagnosed (Aston et al., 2019). In these studies, it is tacitly assumed that admission to the degree programs ensured a high level of subject-specific talent, and the conclusion is therefore that higher education students with demonstrable skills in the arts and engineering are more likely to be dyslexic than students in non-creative subjects.

## A New Framework: Cognitive Search

Approaches to explaining DD must account for both the difficulties and the enhanced abilities that are typical of people with DD. All the proposed strengths outlined above relate in some way to seeking out the unknown, often at the expense of exploiting known information. A useful framework for tying together these observations is cognitive search, which involves a trade-off between exploration–exploitation.

### What Is Search?

Animals need to identify information and resources that have survival value. Since the availability of resources and information varies with time and location, the optimal search strategy will also vary. Moreover, uncertainty caused by environmental variability may obfuscate the optimal strategy. Any search thus involves navigating the trade-off between spending time and energy exploring new possibilities versus exploiting existing information.

Tipping the balance too far toward either exploration or exploitation puts the animal at risk of not obtaining the resources – or knowledge – needed to survive. Exploring endlessly without exploiting what has been found can be inefficient, whereas focusing too much on exploitation may be suboptimal or result in failure to adapt to change. This trade-off arises in many seemingly unrelated areas of endeavor, from evolution to the economy to artificial intelligence (Holland, 1992).

The simplest case is animals foraging for food. They could remain in a known area, where they exploit a local patch of resources; alternatively, they could search globally, exploring the unknown area beyond; or they could pursue any strategy in between. Animals can also search using their sensory systems. In the visual modality, an explorative search strategy could involve taking in a greater proportion of the visual scene to ascertain the visual gist, albeit more diffusely sampled. Another example could be moving the focus frequently between patches of information at the expense of analyzing specific visual points of interest in fine detail.

Search can also occur in more abstract spaces over information landscapes instead of physical ones, e.g., in searching for a new policy or solution to a problem. For problem solving, explorative search would lead to more original solutions rather than the exploitation of solutions that worked in the past. The more globally explored an information space, the greater the possibility of novel recombination or translation of knowledge between realms. Recombination has been argued to be one of the greatest drivers of innovation in the economy and in nature (Arthur, 2009; Page, 2011).

Appropriately balancing the trade-off between exploration and exploitation is essential to adaptive success in a complex, changing world (Cohen et al., 2007), and it is therefore thought to be one of the most significant selective forces operating in the evolution of cognition (Hills et al., 2010). Hence, search can be used as a unifying framework to understand many aspects of cognitive function and behavior across domains (Hills et al., 2015).

Given the nature of the difficulties and strengths proposed to exist in people with DD, we hypothesized that DD may reflect a cognitive specialization toward explorative search. In the next section, we examine whether cognitive and neurophysiological research shows evidence of enhanced explorative search relative to the general population, or any correspondingly diminished ability in local search and exploitation in people with DD.

## Reframing Dyslexia-Associated Cognition From the Perspective of Search

As noted above, search is fundamental to how we understand behavior, from cognitive control over a range of domains to the evolution of cognition. We review existing data on individuals with DD covering a range of different cognitive domains and modalities. We examine evidence for cognitive differences from the perspective of cognitive search as characterized by the exploration–exploitation trade-off. We consider external search (i.e., perceiving and selectively attending to information in the external environment), then internal search (i.e., searching for information in memory or using information from memory to search for solutions to problems), and finally, neurophysiological characteristics. Note that different terms are used across disciplines to refer to an emphasis on exploration or exploitation (see Table 1).

**TABLE 1:** Terminology used to describe exploration and exploitation across fields.

Exploration	Exploitation	Source
Search, variation, flexibility, experimentation, discovery, and innovation	Refinement, choice, production, efficiency, selection, and implementation	(March, 1991) (*Organizational Research*)
Global	Local	(Todd et al., 2012) (*Memory*); (von Károlyi, 2001) (*Visual*); (Williams and Casanova, 2010) (*Minicolumn circuitry*)
Extensive	Intensive	(Benhamou, 2007) (*Foraging*)
Divergent	Convergent	(Martín-Brufau and Berná, 2021) (*creativity studies*); (Hommel, 2012) (*Action-control styles)*
Diffuse	Focused	(Rivière et al., 2017) (*Visual attention*); (Geiger et al., 2008) (*Auditory attention*)
Breadth	Depth	(Korf, 1985) (*Artificial Intelligence*)
Gist	Verbatim	(Reyna, 2005) (*Fuzzy-trace theory of memory*)

Supporting evidence varies greatly, depending on the area of cognition under study and its perceived relevance in understanding reading and writing difficulties. Nevertheless, regardless of the modality or specific terminology used, the recurring pattern that emerges supports the hypothesis that people with DD can be viewed as being specialized in explorative (global) search.

### External Search

Just as organisms search their external environments by moving in physical space, they may also search for external information through attentional search (Hills and Dukas, 2012). An organism’s external world can be imagined as a multi-dimensional search space maintained using information available from all its senses (Hills and Dukas, 2012). Although much irrelevant information may be filtered out, it may still be impossible to process all information relevant to adaptation owing to limitations in sensory processing capacity and the brain’s limited rate of processing information. Given these constraints, a strategy for directing attention toward the most relevant cues in the information space at any particular time is necessary (Hills and Dukas, 2012). The most relevant information is that which confers a survival advantage (Dukas and Ellner, 1993). Hence, similarly to how search may take place in a physical space, in an information space, animals also need to navigate the exploration–exploitation trade-off. Below we consider information search in the visual and auditory modalities to examine how individuals with DD navigate this search’s trade-off in perceptual information spaces.

#### Visual Search

Visual information search refers to the analysis of visual information in order to identify visuospatial characteristics. The existence of visuospatial talents in individuals with DD has often been proposed (e.g., Geschwind, 1982; West, 1997; Eide and Eide, 2011). Gilger reviewed studies of dynamic and complex spatial processing in participants with and without DD, and he found that the empirical data for a general visuospatial advantage were inconsistent, with DD individuals performing a range of visuospatial tasks either as well as or worse than individuals without DD (Gilger et al., 2016). The exception was in the realm of holistic processing, in which individuals with DD consistently demonstrated enhanced abilities (Gilger et al., 2016).

This advantage was first demonstrated in studies using impossible figures, such as Escher’s famous Waterfall. These figures are locally congruent while globally impossible; to detect their impossibility, they must be scanned globally rather than locally (von Károlyi, 2001). Von Károlyi and colleagues found that participants with DD were able to detect impossible figures significantly faster than non-DD participants without a loss of accuracy (von Károlyi, 2001; von Károlyi et al., 2003). They proposed that these results suggest an enhanced ability in rapid and accurate holistic inspection whereby visual spatial information is processed globally rather than locally.

Similarly, individuals with DD have been shown to be faster at 3D mental rotation and manipulation than those without DD (Attree et al., 2009; Wang and Yang, 2011). These results suggest that individuals with DD have access to a unique way of processing visual information (Gilger et al., 2016), a proposal that is consistent with fMRI studies showing that individuals with DD use different functional networks during such tasks (Diehl et al., 2014). Gilger speculated that such a unique mode of information processing might also yield advantages in other tasks that require unique perspective-taking or an ability to see patterns in a distracting context of complex forms.

The notion that DD involves a visual component is long-standing. Research looking at more fundamental aspects of visual processing further supports the view that individuals with DD process information more globally as a trade-off for decreased local processing. Several studies have found that individuals with DD have deficits in focal attention (Facoetti et al., 2008; Ruffino et al., 2010) but better resolution for features in the periphery of the visual field (Geiger and Lettvin, 1987; Perry et al., 1989; Lorusso et al., 2004). This includes enhanced perception of low-spatial-frequency components, that is, features such as global shape, as opposed to high-spatial-frequency features such as sharp edges and fine details (Schneps et al., 2012). In contrast, it has been noted that non-DD individuals are more adept at identifying details located in the center of the visual field (Geiger and Lettvin, 1987; Perry et al., 1989; Lorusso et al., 2004).

As an alternative to the magnocellular deficit theory (Stein and Walsh, 1997; Stein, 2001, 2019), Schneps et al. (2012) posited the theory that there is instead a magnocellular shift toward the periphery in people with DD, according to which magnocellular density is reduced at the fovea and enhanced at the periphery. Schneps et al. (2007) proposed that people differ in the extent to which they can make use of information in the central versus peripheral fields, with these differences in turn affecting their tendencies for focused search versus broad comparisons. Taken together, these studies indicate that individuals with DD have lesser abilities in local visual search (exploitation) and enhanced abilities in global (explorative) visual search.

#### Auditory Search

Compared with visual search, less attention has been paid to DD-associated auditory differences from the perspective of search, but a study by Geiger et al. (2008) provided some insights. They investigated whether children with and without DD differ in their abilities on an auditory task. The task involved perceiving a set of stimulus words from a central location, first without interference, and then under two different masking conditions (white noise and a “cocktail party” speech mask) creating interference from the periphery.

For both groups, recognition performance was comparable in the central non-interference condition. However, in the cocktail party condition, the group with dyslexia performed significantly worse (Geiger et al., 2008, 3A). Their inferior performance seems to be associated with higher recognition intrusions from the speech masker (Geiger et al., 2008, 3B), indicating an inability to disregard the peripheral speech. This pattern mirrored findings for a companion task in the visual domain, leading Geiger et al. (2008) to suggest that individuals with DD have a wider spatial attention than those without DD in both auditory and visual modalities.

### Internal Cognitive Search

Humans also engage in internal cognitive search for information stored in memory, retrieving or internally manipulating such information to search for solutions. This section discusses different areas of memory and memory paradigms and how they relate to cognitive search. Evidence reviewed from a range of studies lends further support to the hypothesis that individuals with DD demonstrate a bias toward explorative internal search.

#### Procedural Memory

Procedural memory is a long-term memory system involved in implicit learning and use of knowledge; that is, memory that is not available to conscious awareness (Squire, 2004). Procedural memory supports learning and the execution of motor and cognitive skills, particularly those involved in sequences, and it engages a wide network of specific frontal, basal-ganglia, parietal, and cerebellar structures (Ullman, 2004). Learning to read, write, or play the piano are all examples of skills that are dependent upon procedural memory; once learned, the skills can be processed automatically and rapidly (Lum et al., 2013). Individuals with DD have been shown to be less efficient at procedural learning than non-DD individuals (Lum et al., 2013). It has been proposed that many of the difficulties observed in DD individuals may be explained by a failure to automatize skills because of an impaired procedural memory system and the underlying deficits thought to exist in the cortico-cerebellar circuit (Nicolson and Fawcett, 1990, 2007; Nicolson et al., 2001).

Automaticity allows tasks to be executed more quickly and efficiently. However, from the perspective of cognitive search, once a skill becomes automatic, one is essentially exploiting the same information again and again. Conversely, if an individual has difficulty acquiring automaticity, they retain declarative (conscious) awareness of the process. Therefore, they can still explore new, potentially better strategies, and integrate knowledge with other declarative information as it becomes available (Nicolson, 2014). This way of processing information may be slower and more effortful, but the trade-off is that it facilitates explorative search.

#### Fuzzy-Trace Theory

The exploration–exploitation trade-off in cognitive search also aligns with the trade-off present in the fuzzy-trace theory (FTT) of memory encoding and retrieval. FTT posits that information is represented in two parallel, independent memory traces called verbatim and gist (Reyna, 2005). Whereas verbatim traces encode literal information that supports precise analysis, such as the order of letters in a word or the digits in a number, gist traces are fuzzy but meaning-based representations such as context or category. While verbatim processing does not consist of meaningful interpretation, gist is characterized as insightful intuition (Brust-Renck et al., 2021).

This distinction between verbatim and gist trace memories resembles the contrast between local and global cognitive search. Local search is thought to involve the identification of between-item similarity, whereas in global search items are activated when they are related by context or category (Todd et al., 2012). People with DD have been shown to use synonyms more often when they fail to recall the exact form of a sentence compared with those without DD (Miles et al., 2006). This result attests to intact semantic representations in DD, and Obidziński and Nieznański (2017) have suggested therefore that individuals with DD may have enhanced gist memory. They used multinomial models to measure verbatim and gist memory processes and found that individuals with DD have poorer verbatim trace memory than participants without DD. However, they also reported higher probability of gist trace retrieval when semantically similar stimuli were presented to individuals with DD compared with controls. These results are relevant in the context of global search, as explained above, where items are activated in relation to the overall category. Thus, deficits in the cognitive process that facilitates differentiation between orthographically similar items may be accompanied by an enhanced ability to recognize semantic similarity.

#### Divergent Thinking

Several studies have shown that individuals with DD have enhanced abilities in various aspects of divergent thinking (Cockcroft and Hartgill, 2004; Akhavan Tafti et al., 2009; Bigozzi et al., 2016; Kapoula et al., 2016; Lam and Tong, 2021). Divergent thinking includes the ability to generate many solutions or ideas to solve a problem (fluency), flexibility in switching between categories, and the ability to elaborate and develop an idea. It also includes originality, i.e., the capacity to produce novel and unusual ideas (Furley and Memmert, 2015), which is a central feature of creativity (Runco and Acar, 2012). In contrast, convergent thinking “typically leads to conventional and “correct” ideas and solutions rather than original options” (Runco and Acar, 2012, 66). Regarding search, the cognitive control of explorative behavior is likely to require a divergent decision-making style, in contrast to exploitation which relies on a more convergent style (Hommel, 2012). The relationship between external search and divergent thinking was investigated by Martín-Brufau and Berná (2021), who found that high explorative external search ability corresponded to greater divergent-thinking ability; a relationship which they argued reflects shared mechanisms.

Several studies have found that individuals with DD significantly out-perform their peers on various aspects of divergent thinking (e.g., Cockcroft and Hartgill, 2004; Akhavan Tafti et al., 2009; Bigozzi et al., 2016; Kapoula et al., 2016). It should be noted that such studies often focus on non-verbal tests of creativity to avoid literacy confounds (e.g., Cockcroft and Hartgill, 2004; Bigozzi et al., 2016). Indeed, when verbal, figural and non-verbal tests of creativity were used, Lam and Tong (2021) found that children with DD performed worse on verbal creativity, equally on figural creativity but they out-performed their peers on non-verbal creativity. A meta-analysis similarly found that groups with dyslexia showed a significant performance disadvantage in verbal versus figural creativity (Erbeli et al., 2021a).

Bigozzi et al. (2016) and Akhavan Tafti et al. (2009) found that in fluency and flexibility subtests, the performance of participants with DD was equivalent to their peers, whereas in other studies performance was significantly better (Cockcroft and Hartgill, 2004; Kapoula et al., 2016; Lam and Tong, 2021 (in non-verbal tasks)). Most studies found that individuals with DD significantly outperformed those without DD in tests of originality (Cockcroft and Hartgill, 2004; Akhavan Tafti et al., 2009; Bigozzi et al., 2016; Kapoula et al., 2016; Lam and Tong, 2021 (in non-verbal tasks)), although this result may not always be consistent (Majeed et al., 2021). Bigozzi et al. (2016), who differentiated between fluency and originality to avoid possible confounding factors, still found greater originality among those with DD. Such findings align with the results of another study which showed that students with DD performed significantly better on tasks that involved connecting unusual combinations of ideas supporting new possibilities and original solutions (Cancer et al., 2016). Two recent meta-analyses highlight that a consistent creative advantage is not always found in children and adolescents with dyslexia (Erbeli et al., 2021a; Majeed et al., 2021). However, Erbeli et al. (2021a) found that compared with adolescents, adults with dyslexia did show a creative advantage over non-dyslexic adults. Initial evidence also suggests that enhanced creativity in individuals with dyslexia may be more pronounced in females than males (Erbeli et al., 2021a). Majeed et al. (2021) also found that in the adult samples, individuals with dyslexia significantly out-performed those without dyslexia on creativity scores.

#### Episodic Memory

Differences in declarative memory have also been proposed to exist in individuals with DD. Declarative memory supports the encoding, storage, consolidation, and conscious recollection of factual knowledge (semantic memory) and personally experienced events (episodic memory) (Squire, 2004; Lum et al., 2013).

From an evolutionary perspective, the utility of memory is to guide and influence future actions. Semantic memory is concerned with knowledge that is not tied to its context of acquisition, such as facts (Tulving, 2002; Moscovitch et al., 2016) and which, we suggest, might be viewed as supporting more local search. By contrast, episodic memory encodes the context of past experience, including information specific to the time and space of acquisition (Tulving, 2002). A key adaptive function of episodic memory is to also allow individuals to flexibly retrieve and recombine these building blocks of previous experiences to envisage future events (Schacter and Addis, 2007; Schacter et al., 2017). Hence, episodic memory supports more explorative search through imagined simulations and future outcomes (Hills et al., 2015), referred to as episodic future thinking (Atance and O’Neill, 2001).

Episodic future thinking provides an internal search space, a simulation of future possibilities, through which one can search to explore and evaluate possibilities. It allows one to predict the likelihood of a future outcome even for possibilities that have not been experienced previously (Buckner and Carroll, 2007; van der Meer et al., 2012; Schacter et al., 2017). As such, it saves time and energy, avoiding the need to physically explore different possibilities and enabling an individual to anticipate and avoid problems. However, it also delays action, which could carry risks in itself (Hills et al., 2015). Time spent searching internally is also therefore subject to the trade-off between exploration and exploitation (Hills et al., 2015).

Eide and Eide (2011) proposed that individuals with DD have enhanced episodic memory and that they rely preferentially on episodic rather than semantic strategies for long-term memory. This proposal aligned with later research which found that, in the general population, individuals differed in how they remembered the past – some had richer episodic memory while others more readily retrieved the semantic features of events (Sheldon et al., 2017).

Studies of declarative memory in individuals with DD have yielded inconsistent results, possibly reflecting the use of verbal tests that tax cognitive abilities known to be weaker in people with dyslexia (Hedenius et al., 2013). To avoid such problems, Hedenius et al. (2013) tested object recognition after incidental encoding since incidental learning rather than intentional encoding, and recognition rather than free recall, are less reliant on working memory and executive function. Finding enhanced recognition scores in individuals with DD, they speculated that the advantage in declarative memory might be mediated by a compensatory seesaw interaction derived from a deficit in procedural memory. Attree et al. (2009) also employed an incidental-learning paradigm and studied spatial memory in DD using a realistic computer-generated virtual environment. Under these conditions, the group with DD also scored higher compared with a non-dyslexic control group.

The amount of detail revealed in episodic simulations (i.e., imagined events) has been shown to be strongly correlated with the level of detail retrieved from episodic memories (Addis et al., 2016). Moreover, studies using episodic specificity induction (ESI), whereby subjects are briefly trained to recollect more details from episodic memories, found an increase in the detail of subsequent episodic simulations (Madore et al., 2014). Conversely, studies indicate that limitations in an individual’s ability to recall details of past experiences correspond to limitations in the ability to generate detailed simulations of future possibilities (see Szpunar and Radvansky, 2016, 211).

Studies of amnesiac patients for example have found that deficits in episodic memory positively correlate with an impoverished ability to construct new imagined experiences (Hassabis and Maguire, 2007; Race et al., 2011). It is therefore predictable that, if individuals with DD have enhanced episodic memory, they should have correspondingly enhanced episodic future thinking abilities. Having the ability to create richer internal simulations may facilitate explorative search for successful solutions.

In terms of convergent versus divergent thinking abilities (described in the preceding section), ability in future simulation is expected to be related to the latter but not to the former (Addis et al., 2016). Addis et al. (2016) showed that greater divergent-thinking abilities are associated with greater capacity to imagine more detailed future episodes. In addition to increasing simulation detail, ESI has been shown to enhance divergent thinking (Madore et al., 2015).

This link between episodic memory, episodic simulation, and divergent thinking is further supported by a study in which amnesic patients with diminished episodic memory for past experiences had difficulty imagining the future as well as decreased abilities in divergent thinking (Duff et al., 2013). This finding is consistent with fMRI evidence (Benedek et al., 2014) showing that “divergent thinking recruits some of the same default network regions typically linked with future simulation” (Addis et al., 2016, 95). In addition to evidence for enhanced episodic memory, greater divergent thinking ability in adults with DD, therefore provides further supporting evidence for Eide and Eide’s (2011) proposal that people with DD have enhanced episodic memory, and in turn enhanced ability in explorative search through episodic future thinking.

#### Working Memory

Working memory (WM) refers to the ability to process information and store the intermediate products of that processing for a brief period before using it (Ghani and Gathercole, 2013). It is well established that individuals with DD typically have a low WM capacity with regard to the central executive and phonological loop (Ghani and Gathercole, 2013) as well as the visuospatial sketch pad (Lipowska et al., 2011).

The role of cognitive search strategies in memory has been investigated in studies of the relation between phonological WM capacity and how information is retrieved from long-term memory (Rosen and Engle, 1997). Local search is thought to involve looking for similarity between items, whereas in global search items are activated in relation to the context and overall category (Todd et al., 2012). Individuals with a high WM capacity have been shown to transition less frequently between local and global cues compared with individuals with a low WM capacity (Rosen and Engle, 1997; Hills and Pachur, 2012). This indicates that individuals with high WM capacities are better at exploiting local information when searching in long-term memory while individuals with a lower WM capacity, (such as individuals with DD), tend to move more readily from local patches of information to global exploration (Todd et al., 2012). These findings agree with observations of individuals with DD found to be particularly talented at seeing “relationships of likeness and “togetherness”; connections between perspectives and fields of knowledge; and big-picture or global connections that create heightened abilities in detecting gist, context, and relevance” (Eide and Eide, 2011).

Several studies have observed a negative correlation between WM capacity and divergent-thinking ability. For example, training WM using a mental calculation paradigm has been found to improve WM capacity but reduce performance in divergent-thinking tasks (Takeuchi et al., 2011). While none of the studies have looked at DD, some have considered ADHD, a diagnosis frequently given alongside dyslexia (Germanò et al., 2010). In studies of cognitive search, ADHD is considered a pathology related to goal-directed search, characterized by too much exploration (Hills, 2006; Todd et al., 2012). Individuals with ADHD have been shown to have a low WM capacity (Kofler et al., 2010; Rhodes et al., 2012; Fugate et al., 2013) and better performance in aspects of creative thought such as conceptual expansion and the ability to overcome knowledge or example constraints (Shaw, 1992; Abraham et al., 2006; White and Shah, 2016). They also score more highly on originality in divergent-thinking tasks (White and Shah, 2006, 2011).

Fugate et al. (2013) studied divergent-thinking ability in students with high fluid intelligence who were diagnosed with ADHD. They had speculated that low WM could reduce the ability to form novel combinations of information as it would reduce the ability to hold information in mind. Instead, they found that the lower the WM capacity, the higher the levels of divergent thinking (Fugate et al., 2013). Not all studies have found a negative correlation between WM and divergent-thinking ability, so other factors such as intelligence or processing speed may also play a role (Takeuchi et al., 2020).

In addition to the ability for divergent thinking, lower WM has also been associated with enhanced insight-based reasoning (DeCaro et al., 2016). Insight refers to the sudden reinterpretation of a stimulus, situation, or event to produce a non-obvious interpretation, seemingly disconnected from the stream of conscious thought, that finds a solution to a problem or the comprehension of a joke or metaphor (Kounios and Beeman, 2014). Insight-based reasoning contrasts with deliberate, analytic, incremental problem solving, which is also associated with different patterns of brain activity (Kounios and Beeman, 2014). Given these characteristics, insight-based reasoning may be considered as a more explorative process and analytical reasoning of a more exploitative one.

Although there have been no formal studies of reasoning style in DD, insight-based reasoning has been proposed, based on clinical observations, as an area of enhanced ability (Eide and Eide, 2011). Support for this proposal came from later experimental work revealing that high WM capacity has a negative impact on the ability to perform the problem restructuring and solving processes necessary for insight (DeCaro et al., 2016). This is thought to be because insight problem solving relies on “associative processes that operate outside of close attentional control” (DeCaro et al., 2016).

Lower WM capacity is typically viewed as a shortcoming in people with DD compared with people without DD. However, available evidence on WM also suggests that those with DD might experience compensatory advantages in their capacities for divergent thinking and insight-based reasoning, that is, in cognitive domains manifestly related to explorative search.

Similarly, the pattern emerging from evidence related to internal cognitive search is that DD-associated cognition shows both a diminished ability to exploit and, generally, a correspondingly enhanced ability to explore.

### Neurophysiological Differences

#### Minicolumn Circuitry

In addition to cognitive differences, there is also evidence for neurophysiological differences in individuals with DD that relate to the exploration–exploitation trade-off. One such difference regards minicolumn circuitry. Minicolumns are an elementary unit in the neocortex of all mammalian brains (Buxhoeveden and Casanova, 2002). They are essential in cortical information processing, with differences in connectivity within and between modular cortical circuits relating to differences in how information is processed (Casanova and Tillquist, 2008; Williams and Casanova, 2010). In a study by Williams and Casanova (2010), the minicolumn circuitry for individuals with DD was found to have stronger global connectivity at the cost of local connectivity relative to controls and individuals on the autism spectrum. Specifically, greater mini-columnar width and spacing and fewer minicolumns result in fewer local connections. The corresponding enlargement of the gyral window makes a larger number of commissural fibers possible, increasing in turn the volume of tracts such as the corpus callosum (Williams and Casanova, 2010). Thus, decreased local connectivity in the cortex benefits long-range connectivity.

Williams and Casanova found the inverse to be true of individuals on the autism spectrum, who were found to have stronger local connectivity. In this case, relative to controls and individuals with DD, individuals with autism have a greater number of minicolumns with reduced width and reduced spacing, enabling hyperconnectivity in short-range connections within these modules. Furthermore, an associated decrease in the size of the gyral window places constraints on the developing commissural white matter and contributes to a decrease in long-range connectivity between modular units.

Williams and Casanova proposed that these differences in minicolumn circuitry give rise to a spectrum of cognitive styles that range from those with a holistically oriented, gestalt processing bias (DD) to those with a detail-oriented or local processing bias (autism). In other words, owing to physical limitations in the brain, individuals with DD have a global processing bias resulting in enhanced abilities for exploring information, and vice-versa for individuals with autism. These results align with the pattern that individuals with DD have a global search bias.

## Are Individuals With Developmental Dyslexia Specialized in Explorative Search?

We have considered research on DD from the perspective of cognitive search as characterized by the exploration–exploitation trade-off. Since the differences observed in people with DD were initially observed because of difficulties found in reading and writing, studies to date have primarily focused on areas of cognition relevant to understanding these difficulties. Despite this focus, when taken as a whole, the evidence reviewed shows a pattern of stronger explorative search specialization (global search) for those with DD, with the corresponding trade-off in weaker exploitation (local search). Here, we consider additional lines of evidence that are relevant to this more specific proposal of search specialization.

### Explorative Bias Found at All Levels of Analysis, Internal and External

The explorative bias for people with DD can be seen at multiple levels of analysis. Proposed strengths cluster around explorative behaviors such as big-picture, long-term thinking and inventiveness. Were cognition to show a local processing bias in individuals with DD, one might infer that the behavioral differences they appear to exhibit do not reflect explorative specialization but rather, are coping strategies they’ve developed, perhaps in the face of adverse educational experiences; that is, the apparent explorative strengths associated with DD would be circumstantial. However, this is not the case: instead, an overall bias toward explorative cognitive search is seen across multiple areas of cognition. Furthermore, in line with Hills et al. (2008), search preferences in the external domain generalize across to internal search domains; i.e., a global search bias is found in visual and auditory search (external) as well as in memory search (internal). At a more fundamental level, an explorative bias in minicolumn circuitry is observed: individuals with DD exhibit greater global connectivity at the expense of local connectivity (Williams and Casanova, 2010). This pattern of explorative specialization emerges from the data, even though the studies were undertaken in different domains by researchers who were not considering the perspective of search.

### Functional Constraints

In the context of an already cooperating group, within-species division and specialization is favored when features that confer fitness benefits are functionally incompatible (Rueffler et al., 2012). Since the human brain has limited capacity, enhanced abilities in particular functions or systems may come at a trade-off to capability in other processes and systems (Hofman, 2001; Marois and Ivanoff, 2005; Colzato et al., 2022). In keeping with this principle, it has been proposed that during human evolution, once a certain level of proficiency had been reached, the only efficient way to increase cognitive search effectiveness was through specialization (Taylor et al., 2022).

The existence of such trade-offs has long been reported in individuals with DD (Eide and Eide, 2011), and such instances have been highlighted throughout this paper. For example, physiological constraints related to minicolumn circuitry create a trade-off between global versus local connectivity. In another realm, a high level of WM comes at a cost to processes required for insight-based reasoning (DeCaro et al., 2016). In other areas of internal search, Hedenius et al. (2013) highlighted the possibility of competitive interactions between declarative and procedural memory. Within procedural memory, trade-offs exist between the benefits of automaticity and the opportunity to innovate (Nicolson, 2014). The functional constraints and competitive interactions that preclude enhanced ability in search beyond a certain level, support the proposal that individuals with DD are specialized in explorative search. Notably, many physiological and cognitive markers for DD risk precede exposure to reading (Ozernov-Palchik and Gaab, 2016). If such diminished abilities are trade-offs that exist to enable enhanced abilities in exploration, this would lend further support to the notion that explorative abilities also exist before educational difficulties arise, and are innate.

### Prevalence Rates

We have already noted the very high prevalence rate of 5–20% (Badian, 1984; Wagner et al., 2020) and the at least 60% heritability (Paracchini et al., 2016) of DD in the general population, supporting the notion that DD-associated cognitive differences play an important role. Division and specialization, specifically in search, is also found in other organisms. Although phylogenetically distant, humans share many characteristics with social insects (Crespi, 2014), many species of which have also evolved to specialize in different search strategies. Interestingly, in bees, the proportion of scouts (i.e., those individuals with an explorative search bias) that make up a colony’s foraging force is around 5–25% (Liang et al., 2012), comparable to the 5–20% prevalence of DD in humans (Badian, 1984; Wagner et al., 2020). Here we can only use reading and writing difficulties as a proxy for the proportion of the human population with an explorative search specialization, and, as noted earlier, this is a continuous trait with no clear cut-off point. Nevertheless, the comparison with social insects serves to highlight the point that in other species we recognize the existence of search specialization. The high prevalence rate in humans also implies that, rather than a dysfunction or an aberration of search, DD reflects a specialization of search behavior.

### Explorative Cognition Correlates With Environmental Variability During Human Evolution

Why did specialization in search evolve, what kind of selection pressures would have led to this, and did these pressures exist during our evolutionary history? Specialization in search is likely to have been selected for by a highly variable and uncertain environment (Taylor et al., 2022). In uncertain environments, continual exploration ensures that exploitation remains optimal. Furthermore, owing to the functional constraints discussed above, there is a certain threshold at which enhanced ability in exploration comes at the cost of abilities to refine and to exploit. Specialization would suggest that, over evolutionary time, there were strong selection pressures to excel in search and adaptation to an extent beyond the capabilities of single individuals. This suggestion leads to the testable prediction that the environmental conditions shaping the evolution of human cognition were extremely uncertain and variable. In terms of when we would expect this variability to have occurred, the cross-cultural nature of DD makes it possible to infer that the associated brain differences were selected for prior to behavioral modernity, suggesting a latest possible date of 50,000 BP.

**FIGURE 1:**
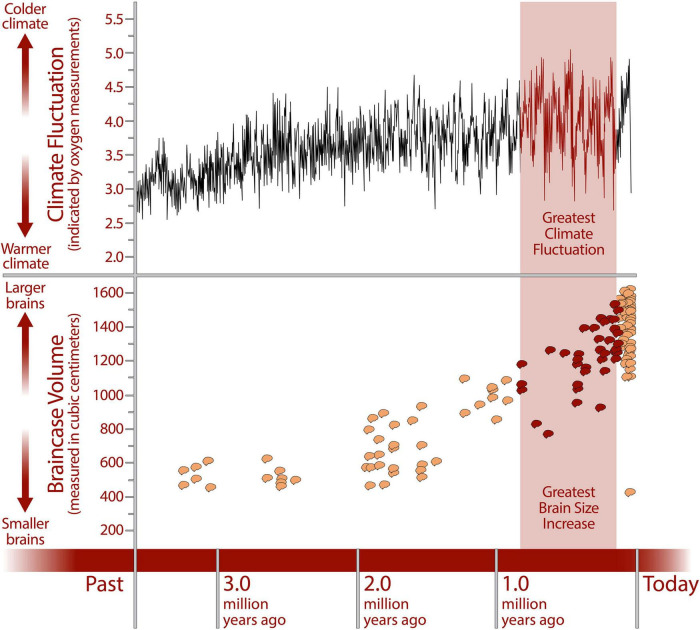
Lower graph showing the increase in cranial capacities of hominin fossils over the last 2 million years, indicating an increase in brain size, primarily within the genus *Homo* (Potts, 2011). Cranial capacity data (Holloway et al., 2004; Falk et al., 2007; Carlson et al., 2011). The upper graph shows Earth’s climate fluctuations during the same period (data from Zachos et al., 2001) including the strongest fluctuations, which coincided with the period of greatest brain size increase. Credit: Human Origins Program, Smithsonian Institution.

In the field of paleoarchaeology there is already a large body of evidence that human evolutionary history was shaped over hundreds of thousands of years by extremely high levels of environmental variability (Potts, 1998; Potts and Faith, 2015) with the period of greatest fluctuation coinciding with the period of greatest increase in brain size (Potts, 2011; Figure 1). Environmental variability was such an important factor in human evolution that it has been argued that, rather than being adapted to a particular environmental context, humans are adapted to variability itself; this is what Potts (1998) has called “variability selection.” Significantly, increased capacity and efficiency in cognitive search arising from specialization and collaboration enables precisely this: an increased capability to adapt and survive, not in one particular environment, but in a range of habitats (Taylor et al., 2022).

## Discussion

People with DD are currently classed as having a neurobiological or neurodevelopmental disorder (Bishop and Rutter, 2008; Valdois, 2010; American Psychiatric Association, 2013; Thapar and Rutter, 2017); this implies that brain development is disrupted in some way, leading to abnormalities and a dysfunctional brain.

In the introduction, we outlined several factors that suggest this form of cognition may instead have an evolutionary basis. First, DD’s high prevalence within the population along with its high heritability imply that DD may provide advantages that complement its better-studied disadvantages. Second, writing is a very recent technology, and the need to read and write is unlikely to have exerted any significant evolutionary selection pressure. Finally, DD has been shown to be strongly polygenic in nature (Erbeli et al., 2021b). In combination with DD’s prevalence and heritability, these factors support the notion that, rather than representing a disorder, DD-associated cognitive differences were selected for and confer some kind of fitness advantage.

We then highlighted that the various DD-associated proposed strengths are all fundamentally related to exploration: global abstract and spatial reasoning, inventiveness, dynamic reasoning (the ability to simulate and make predictions about the future or about the unwitnessed past), and so on. These observations have been consistently highlighted in the literature by practitioners (e.g., Geschwind, 1982; West, 1997; Stein, 2001; Eide and Eide, 2011; Nicolson, 2014; Schneps, 2014). While some have argued that creative abilities in DD are coping strategies rather than inherent capacities, we have outlined multiple lines of evidence that show this is not the case.

The behavioral strengths that have been proposed are unified by the pattern of exploration. This is especially conspicuous considering that the various practitioners who noted these different strengths had neither an awareness nor expectation that DD would correlate with strength in exploration. Furthermore, this shared pattern is particularly striking since it is not immediately obvious that talents such as global spatial reasoning and dynamic reasoning share the same fundamental pattern, however this pattern does become clear through the lens of search. Similarly, incidence studies show particularly high numbers of people with DD in areas of study or work that require explorative ability, e.g., artists, designers, engineers, and entrepreneurs. It is this shared pattern which motivated the hypothesis that individuals with DD are specialized in explorative search. We investigated this by re-examining the extant cognitive psychology and neuroscience evidence from the perspective of search. A pattern of explorative specialization across domains and at all levels of analysis, strongly suggests that the higher-level explorative abilities that have been observed, emerge from these fundamental brain level differences. While more study is required in each area, collectively, an overall pattern of exploitative weakness and exploratory strength emerges.

We also considered a range of other perspectives to examine the possibility that humans (including those with DD) are individually specialized in search. On the one hand, the same explorative bias pattern for those with DD is found at all levels of analysis, including behavioral, cognitive (including internal and external search) and brain level differences. On the other hand, the existence of functional constraints that limit increased capability in search beyond a certain threshold help to explain why specialization may have evolved. The difficulty of optimizing search at the individual level is also reflected in the fact that other social species have also evolved to specialize in different search strategies. Another perspective considered was the alignment of the environmental pressures that shaped human evolution, specifically, “variability selection” (Potts, 1998) with the evidence for cognitive search specialization. The chronological alignment of environmental variability with the likely period at which human search specialization evolved was also discussed. A deeper explanation of the evolutionary theory applied to DD has been presented previously (Taylor et al., 2022). A wide range of evidence assembled from multiple, disparate disciplines points to the same pattern that both associates DD with an explorative bias and explains why this is likely to have evolved.

Taking all these factors into account, the assumption of pathology breaks down. We propose that, taken collectively, these various lines of evidence strongly indicate that individuals with DD do not have a disorder but instead, are specialized in explorative cognitive search.

### Implications for Research on Developmental Dyslexia

The use of the cognitive search framework to unify and explain a wide range of aspects of behavior, cognition, and neurophysiology has implications for future research on individuals with DD and for theories of DD. The dominant approach to DD seeks to understand why individuals with DD have difficulties with the technologies of reading and writing. In viewing DD as a failure to adequately use a certain technology, we have been led to view individuals with DD as abnormal and as having deficits of one kind or another. Ultimately, this assessment is based on a cultural benchmark. The framework of cognitive search used in this article points us toward a new approach for understanding individuals with DD. This approach allows us to ask instead: “what are these brains really built to do?” (Eide and Eide, 2019). Going forward, individuals with DD-associated cognition may be better served by research that adopts the perspective of search rather than the traditional “disorder” or “deficiency” paradigm.

Cognitive search provides a wider, more neutral lens for exploring both cognitive strengths and weaknesses associated with DD. Additionally, it provides a common framework for connecting findings across different areas of cognition, highlighting potentially fruitful avenues for further research. Using the framework of search may also enable new insights regarding reasons for heterogeneity within this population, as well as providing a framework for comparison with other populations. Moreover, it enables us to place DD-associated cognition in a much broader research context, allowing us to understand the importance of this way of thinking when contemplating bigger questions of human adaptation and cultural evolution (see Taylor et al., 2022). Reframing people with DD as specialized in exploration also has implications for how we design educational and academic systems.

### Education and Academia

The current reliance on reading and writing for learning and communication presents problems for individuals whose cognitive abilities favor exploration. The acquisition and use of writing technology appears to align more favorably with individuals who are less exploratory. For example, difficulties characteristic of DD at least partly relate to procedural memory, which, as discussed, enables the exploitation of knowledge. These difficulties arise especially with adapting phonological information and automatizing skills that support reading (Nicolson et al., 2001; Nicolson and Fawcett, 2007). A high level of working memory, also associated with exploitation of information, has been shown to relate to successful acquisition of skills and knowledge in reading (Gathercole et al., 2006). Efficient phonological decoding relies upon precise visual selection of graphemes (Facoetti et al., 2008), which again falls toward the exploitation end of cognitive search. Williams and Casanova (2010) noted that a lower number of local connections in the cortex has been described for individuals with DD. They suggest that this decreases their feature extraction capabilities, which could also cause deficits in phonological processing.

The alignment between reading/writing and more local processing is further supported by studies of people with hyperlexia: a profile opposite to DD, where reading skills are advanced relative to comprehension or general intelligence. Ostrolenk et al. (2017) found that over 80% of those with hyperlexia were also on the autism spectrum, which has been found to be characterized by a strong local search bias (Frith, 1989; Happé and Frith, 2006). This further strengthens the view that a less exploratory search strategy might better support reading and writing abilities. Conversely, some individuals with autism may also suffer reading-related difficulties, but they appear to be opposite and complementary to those found in people with DD. Whereas DD readers may show superior processing for meaning relative to their decoding abilities, autistic readers on average show stronger decoding strategies on a word-to-word basis relative to their abilities in reading comprehension (Frith and Snowling, 1983; Henderson et al., 2014; Snowling et al., 2020).

In summary, these examples show how different theories of DD tend to cite weaknesses in aspects of cognition or neurology that are related to exploitation of information as contributing factors, and how the emphasis on this technology for communication and learning may disadvantage more exploratory individuals.

Similarly, most education and academic systems strongly favor less exploration. Education systems that primarily assess an ability to reproduce information that is known, as opposed to using information to develop new solutions and to explore the unknown, put more explorative individuals at a significant disadvantage. In Western academic systems, reward is based on the quantity of written output, and narrowly specialized local search tends to traditionally be favored over interdisciplinary global search. Thus, although academic research is ostensibly explorative, the cognitive style of explorative academics is generally not rewarded.

Given these factors, it is unsurprising that individuals with a more explorative cognitive style would struggle in academic environments. Activities that are valued and linked to assessment and advancement highlight their weaknesses; at the same time, they are given little opportunity to express and develop their strengths, causing frustration, stress, and anxiety. The sustained emotional toll they bear over an extended period (most of their pre-adult life, if not more) can lead to a variety of harms, including post-traumatic stress disorder as adults (Alexander-Passe, 2015) as well as higher rates of self-harm (Scott, 2004), suicide (Fuller-Thomson et al., 2017), and imprisonment (Moody et al., 2000; Lindgren et al., 2002; Rack, 2005).

The need to balance explorative learning along a continuum with learning through exploitation to reach optimality is well known in other fields such as organizational and machine learning (March, 1991; Holland, 1992). This contrasts with the more narrow view of learning in education where the emphasis is on acquiring (exploiting) existing knowledge. Given the analogous paradigm present in cognition, it would be logical and beneficial to develop and introduce approaches in education and academia that nurture an explorative orientation toward learning (Mulgan, 2021). If as argued humans specialize in search and adapt cooperatively, such changes are even more critical to implement.

### Implications for Research on Other Neurodevelopmental Disorders

Reframing DD from the perspective of search may have implications for understanding other individuals described as having neurodevelopmental disorders. We have suggested that the difficulties experienced by people with DD emerge as a result of a mismatch between their specialization in exploration and demands in educational practices that require a more exploitative processing bias. This suggestion raises the question whether individuals diagnosed with other neurodevelopmental disorders may also be experiencing a mismatch between their cognitive processing abilities and contextual demands rather than simply having deficits. Here we briefly consider ADHD and autism.

An ADHD diagnosis is given alongside DD in 30–50% of cases (Germanò et al., 2010) and there may be some similar underlying mechanisms (Czamara et al., 2013). A genome-wide association study found significant associations between DD and ADHD while no statistically significant genetic correlates were found between dyslexia and autism (Gialluisi et al., 2021: Table 3).

People diagnosed with ADHD have been framed as having a pathology of goal-directed control, an “aberration” of search leading to too much exploration (Hills, 2006; Todd et al., 2012). However, in several studies an ADHD, diagnosis correlates with divergent-thinking ability (White and Shah, 2006, 2011, 2016; Fugate et al., 2013) and more explorative foraging behaviors in both visual and semantic search (Van den Driessche et al., 2019), leading to the proposal that individuals with ADHD characteristics may have a cognitive search strategy that is beneficial in some contexts (Van den Driessche et al., 2019). Furthermore, cognitive differences found in some people diagnosed with ADHD may be regarded as complementary in a way that enhances group performance (Abraham et al., 2006; Zentall et al., 2011). Abraham et al. (2006) found that people diagnosed with ADHD preferred to generate new ideas whereas those without ADHD preferred to develop ideas, exemplifying how different search strategies can work together.

It is important to note however that researchers are increasingly questioning whether diagnostic labels such as ADHD reflect unified groups of people (e.g., Syme and Hagen, 2020; Astle et al., 2022). For example, children are more likely to be diagnosed with ADHD if they are the youngest in their school class suggesting that they are diagnosed due to their comparatively earlier developmental stage (Karlstad et al., 2017; Root et al., 2019; Caye et al., 2020). Individuals who have experienced childhood trauma may also be given an ADHD diagnosis (Szymanski et al., 2011; Brown et al., 2017). Such examples highlight the problem of potentially overly broad catchment when using behavioral diagnostic criteria.

Furthermore, it has been argued that some problematic symptoms of ADHD may be best explained by stress. Stress is experienced when a person feels threatened because they conclude that they are ill-equipped for a task they must perform (Salas et al., 1996). Cotton (2020) found a positive correlation between ADHD severity and a chronic stressor score, where nearly half of possible stressors related to school experiences. Aligning with these results, Syme and Hagen (2020) argued that ADHD characteristics may only be problematic in highly structured, modern contexts such as classrooms and some workplace environments. Stress created by educational or workplace environments might also contribute to understanding why so many people diagnosed with autism also receive an ADHD diagnosis.

Keeping these diagnostic issues in mind, we speculate that at least some individuals diagnosed with ADHD may be specialized to have more explorative search strategies, with more problematic symptoms arising because of cultural practices that induce stress and trauma.

Autism may also be understood from a search perspective. Some types of autism involving stereotypies (repetitive movements or utterances) are framed as pathologies of goal-directed control in terms of exploitation or local search (Hills, 2006; Hills and Dukas, 2012). However, a search perspective may apply more broadly in that many individuals on the autism spectrum can be viewed as having a superiority in local, depth-first search reflective of a processing bias rather than a deficit (Frith, 1989; Happé and Frith, 2006).

This local or depth-first processing superiority may be reflected in different ways such as greater pitch sensitivity (Bonnel et al., 2003); a more detail-focused drawing style (Mottron et al., 1999; Booth et al., 2003), superior performance when identifying geometric shapes in larger complex images (Shah and Frith, 1983; Happé and Frith, 2006), strong memory for facts (Happé and Vital, 2009) enhanced verbatim analytical processing (Reyna and Brainerd, 2011); and ability to master systems that require the discovery of if-then rules and regularities (Baron-Cohen et al., 2002).

Resource-scarce environments characteristic of certain phases of human evolution (Potts et al., 2018) are likely to exert a particularly strong selection pressure for more efficient local cognitive search, as supported by research in patch exploitation theory (Stephens et al., 2012). It has also been proposed that autistic traits, namely a local processing bias, were selected for in such contexts (Spikins et al., 2018). This kind of enhanced depth-first search orientation may play an important role in expanding the band-width of cultural inheritance and increasing capacity for adaptation.

### Implications for Wider Society

Rather than simply focusing on individual cognition, we suggest taking a step back to also consider cognitive search at the group level. Humans primarily adapt through cultural adaptations, exploring, optimizing and consolidating behaviors or inventions across multiple domains that contribute to our survival. It has been proposed that different cognitive search strategies contribute to this knowledge creation process in complementary ways (Taylor et al., 2022). Different cognitive search strategies might currently be viewed as personality differences, or in other cases where they clash with modern cultural practices, misapprehended as neurodevelopmental disorders. However, combining information from different cognitive search strategies has the potential to create mutual and synergistic benefits in the co-creation of cultural adaptations. Such collective intelligence may lay at the core of our species’ exceptional adaptive capability. Redesigning educational and other cultural systems with this understanding in mind may not only better serve individual attainment and self-esteem (Colzato et al., 2022) but may also be vital to society as a whole.

## Concluding Remarks

In diagnosing DD as a disorder, the implicit assumption is that the problem or “deficit” exists within the individual. However, considering DD-associated cognition as a search specialization raises the possibility that the “problem” of DD exists in our cultural assumptions and systems. Our failure to recognize these differences as exploratory specialization can harm individuals with DD by subjecting them to social structures that limit opportunities or promote harm: a form of structural violence (Baber, 2017).

We propose that the cognitive differences observed in individuals with DD are not simply reflective of variation in the population. Rather, the strong clustering between exploratory traits and trade-offs suggest that these differences are part of a pattern of specialization and were selected for during human evolution. Nor do these cognitive differences reflect evolutionary mismatch, i.e., traits that evolved earlier and are now merely vestigial or maladaptive (Tooby and Cosmides, 1992; Li et al., 2018). Rather, we argue that the form of cognition represented by DD plays an essential role in enabling humans to adapt.

Given the high prevalence of DD, this in turn would indicate that humans balance the trade-off between exploration and exploitation through specialization in complementary search strategies. Collaboration between these different strategies would allow more efficient and effective search in the co-creation of cultural adaptations, helping to explain our species’ exceptional adaptiveness. Just as genetic search mediates biological evolution, it has been proposed that complementary cognitive search mediates cultural evolution in humans (Taylor et al., 2022).

It is worth emphasizing that once a system’s components specialize, all of them become interdependent and thus essential. De-emphasizing those parts geared toward exploration tips the system excessively toward refining existing solutions. Cultural change may remain, but it is likely to become progressively less adaptive. Systems that refine existing solutions more rapidly than exploring new ones may be effective in the short-term but are self-destructive in the long-term (March, 1991).

Nurturing different individual cognitive strengths and fostering collaboration would help to realize the synergistic benefits of complementary cognitive search strategies. Removing obstacles to explorative learning, and instead harnessing exploration to increase adaptiveness may enable us to better confront the existential challenges presently facing our species and our planet.

## Data Availability Statement

The original contributions presented in this study are included in the article/supplementary material, further inquiries can be directed to the corresponding author.

## Author Contributions

HT: conceived the theory. HT and MV wrote the manuscript and approved the submitted version.

## Conflict of Interest

The authors declare that the research was conducted in the absence of any commercial or financial relationships that could be construed as a potential conflict of interest.

## Publisher’s Note

All claims expressed in this article are solely those of the authors and do not necessarily represent those of their affiliated organizations, or those of the publisher, the editors and the reviewers. Any product that may be evaluated in this article, or claim that may be made by its manufacturer, is not guaranteed or endorsed by the publisher.
